# Clinical sequelae of gut microbiome development and disruption in hospitalized preterm infants

**DOI:** 10.1016/j.chom.2024.09.012

**Published:** 2024-09-24

**Authors:** Robert Thänert, Drew J. Schwartz, Eric C. Keen, Carla Hall-Moore, Bin Wang, Nurmohammad Shaikh, Jie Ning, L. Colleen Rouggly-Nickless, Anna Thänert, Aura Ferreiro, Skye R.S. Fishbein, Janice E. Sullivan, Paula Radmacher, Marilyn Escobedo, Barbara B. Warner, Phillip I. Tarr, Gautam Dantas

(Cell Host & Microbe *32*, ◼◼◼–◼◼◼; October 9, 2024)

In Figure 1D of the originally published article, diamonds indicating the number of preterm infants with metagenomically detected species for *S. caprae* and *E. faecalis* were incorrect. The article has been updated online and the correct version appears in print. The authors regret the error.

## Figures and Tables

**Figure 1D.**
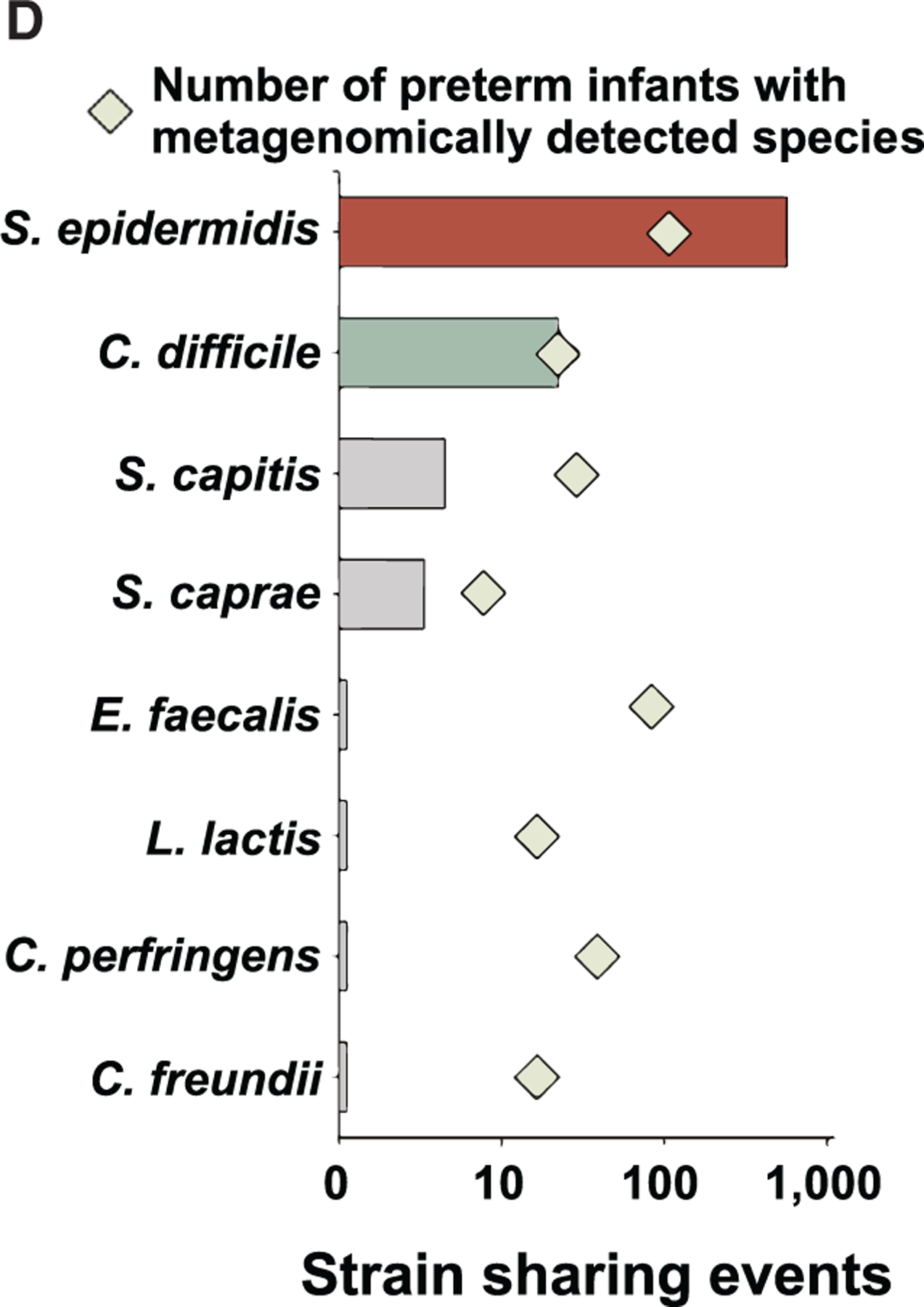
Earliest preterm gut microbiota colonization in the NICU (corrected)

**Figure 1D.**
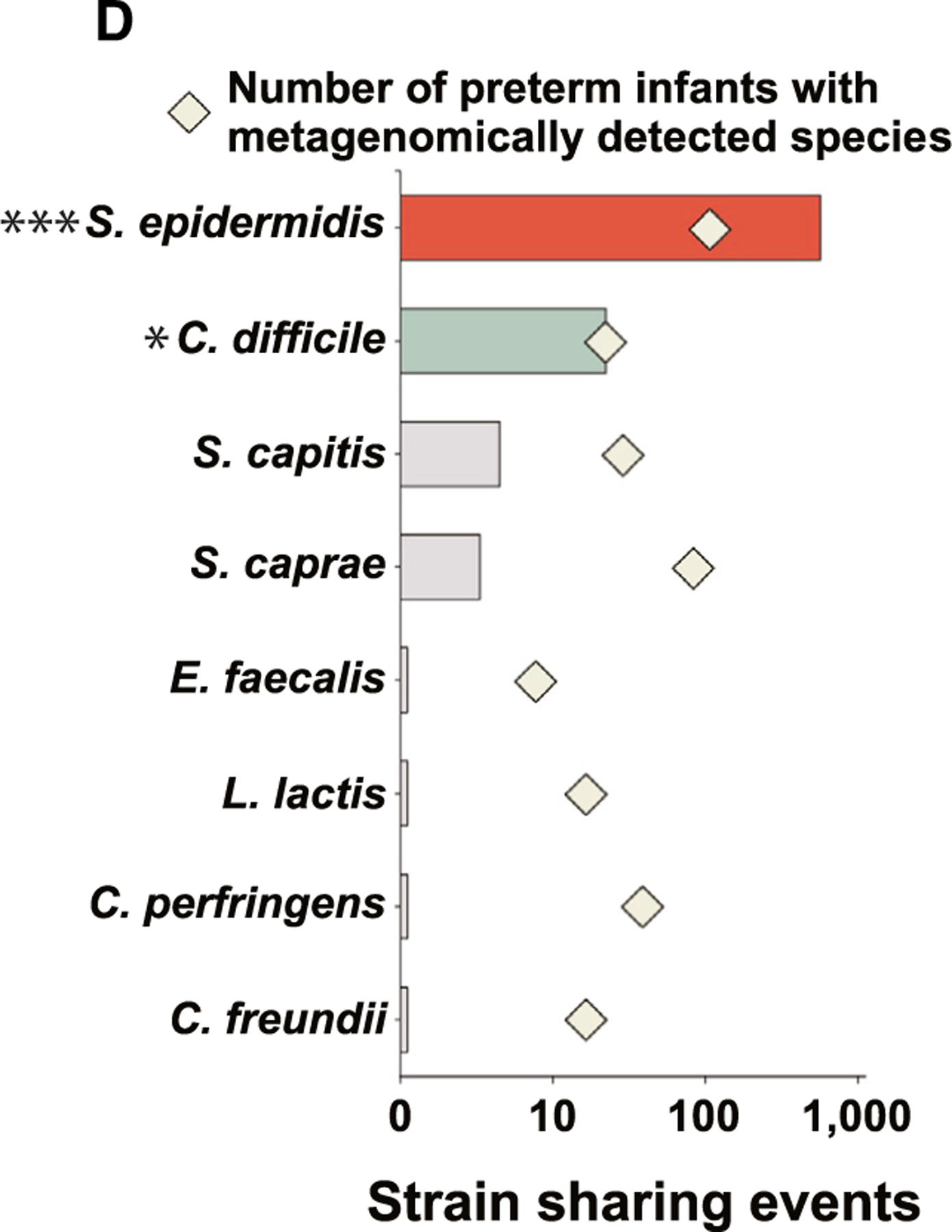
Earliest preterm gut microbiota colonization in the NICU (original)

